# The extracellular SEMA domain attenuates intracellular apoptotic signaling of semaphorin 6A in lung cancer cells

**DOI:** 10.1038/s41389-018-0105-z

**Published:** 2018-12-05

**Authors:** Cheng-Ying Shen, Ya-Chu Chang, Li-Han Chen, Wen-Chun Lin, Yung-Hua Lee, Shu-Tsen Yeh, Hsin-Kuang Chen, Wentao Fang, Chung-Ping Hsu, Jang-Ming Lee, Tzu-Pin Lu, Pei-Wen Hsiao, Liang-Chuan Lai, Mong-Hsun Tsai, Eric Y. Chuang

**Affiliations:** 1 Institute of Biotechnology, National Taiwan University, Taipei, Taiwan; 2 YongLin Biomedical Engineering Center, National Taiwan University, Taipei, Taiwan; 3 Graduate Institute of Biomedical Electronics and Bioinformatics, National Taiwan University, Taipei, Taiwan; 4 Genome and Systems Biology Degree Program, National Taiwan University, Taipei, Taiwan; 5 Department of Thoracic Surgery, Shanghai Cheset Hospital, Shanghai, China; 6 Division of Thoracic Surgery, Taichung Veterans General Hospital, Taichung, Taiwan; 7 Department of Surgery, National Taiwan University, Taipei, Taiwan; 8 Institute of Epidemiology and Preventive Medicine, National Taiwan University, Taipei, Taiwan; 9 Agricultural Biotechnology Research Center, Academia Sinica, Taipei, Taiwan; 10 Graduate Institute of Physiology, National Taiwan University, Taipei, Taiwan; 11 Center for Biotechnology, National Taiwan University, Taipei, Taiwan; 12 Bioinformatics and Biostatistics Core, Center of Genomic Medicine, National Taiwan University, Taipei, Taiwan; 13 School of Chinese Medicine, China Medical University, Taichung, Taiwan

## Abstract

*Semaphorin 6A* (*SEMA6A*), a membrane-bound protein, is downregulated in lung cancer tissue compared to its adjacent normal tissue. However, the functions of *SEMA6A* in lung cancer cells are still unclear. In the present study, full length *SEMA6A* and various truncations were transfected into lung cancer cells to investigate the role of the different domains of *SEMA6A* in cell proliferation and survival, apoptosis, and in vivo tumor growth. *SEMA6A*-induced cell signaling was explored using gene silencing, co-immunoprecipitation, and co-culture assays. Our results showed that overexpression of *SEMA6A* reduced the growth of lung cancer cells in vitro and in vivo, and silencing *SEMA6A* increased the proliferation of normal lung fibroblasts. Truncated SEMA6A lacking the SEMA domain or the extracellular region induced more apoptosis than full length SEMA6A, and reintroducing the SEMA domain attenuated the apoptosis. Fas-associated protein with death domain (FADD) bound to the cytosolic region of truncated SEMA6A and was involved in SEMA6A-associated cytosol-induced apoptosis. This study suggests a novel function of *SEMA6A* in inducing apoptosis via FADD binding in lung cancer cells.

## Introduction

Dysregulation of apoptotic pathways can lead to tumorigenesis through transformation of normal cells to malignant cells^1^. The apoptotic pathways are initiated by death receptors (DRs) on the cell membrane. Whereas, DRs are triggered by their extracellular ligands, molecules such as Fas-associated protein with death domain (FADD) or tumor necrosis factor receptor type 1-associated death domain (TRADD) are recruited to the cytosolic region of the DRs and subsequently activate the downstream death signaling^2^. The loss-of-function mutants of DRs are unable to recruit FADD and are thus inefficient in inducing apoptosis^2^. Furthermore, inactivation of DRs by mutations also shows a high association with some types of cancer, suggesting the importance of DRs in tumorigenesis^3^. However, DRs are not the only apoptosis-initiating proteins. Other membrane proteins like the semaphorin family may participate in regulation of apoptotic signaling^4,5^.

The semaphorin family includes seven subfamilies, all of which contain a characterized SEMA domain. The semaphorin family were initially identified as ligands that control the guidance of axons by directly binding to plexin, thus activating plexin-derived signaling^6–9^. Recently, several studies have demonstrated that the semaphorins have a role in tumor progression. For example, *semaphorin 4D* can act as a pro-tumorigenic factor that induces tumor angiogenesis in head and neck squamous cancer cells^10^. In contrast, several semaphorins, such as the *semaphorin 3* subfamily, function as anti-tumorigenic factors by inducing apoptosis and inhibiting cell proliferation in lung cancer^4,11,12^, breast cancer^5^, and skin cancer cells^13^.

Previously, we observed that semaphorin 6A (SEMA6A), a single-pass transmembrane protein involved in the axonal guidance pathway^14–18^, was significantly downregulated in lung cancer tissues as compared to adjacent normal tissues^19^. Up until now, only a few studies have examined the role of *SEMA6A* in cancer biology^19^, and only one study reported that the extracellular region of SEMA6A could inhibit tumor formation via decreasing VEGF-induced xenograft vascularization^20^. Based on our prior results and another report mentioning that a somatic deletion in *SEMA6A* occurs at locus 5q23.1 in lung cancer cells^21^, in this study, it is hypothesized that *SEMA6A* might play a role in lung carcinogenesis.

Therefore, with the goal of better understanding the importance of *SEMA6A* in lung cancer cells, in vitro cell proliferation, clonogenic and apoptosis assays, and in vivo xenograft animal experiments were performed to examine the functions of *SEMA6A*. Moreover, different truncations of *SEMA6A* were overexpressed in lung cancer cells to study the functions of the parts of *SEMA6A*. Finally, *SEMA6A*-related signaling was delineated by co-immunoprecipitation, co-culture, and gene silencing assays. Through our study we aimed to identify the pleiotropic effects of *SEMA6A* in lung cancer cells, which could be regarded as a potential therapeutic target for lung cancer treatment.

## Results

### SEMA6A is downregulated in lung tumor tissues

**Fig. 1:**
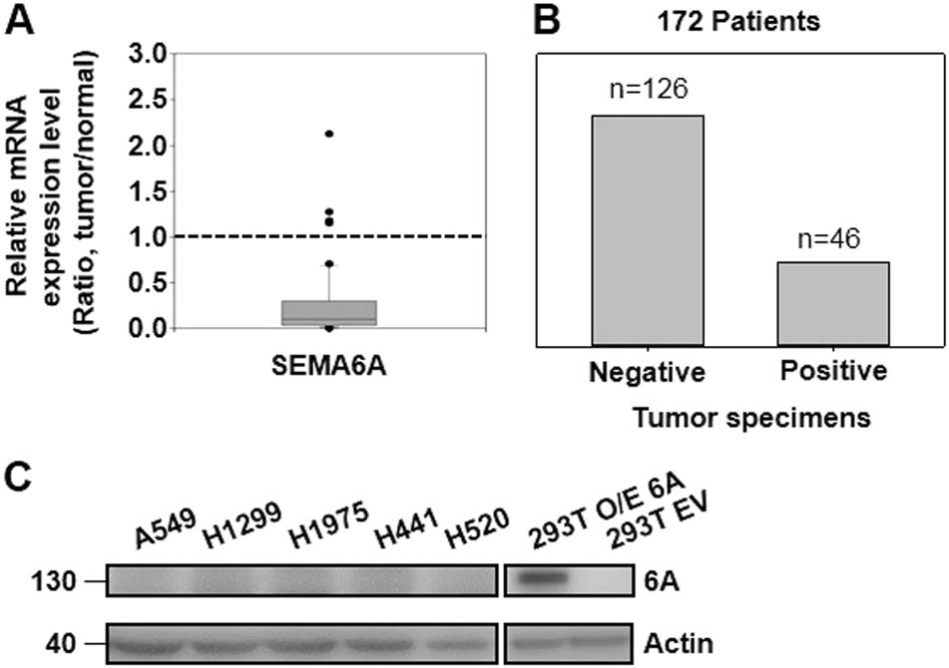
*SEMA6A* is downregulated in lung tumor samples and lung cancer cell lines. **a** Validation of *SEMA6A* expression in lung adenocarcinoma samples (*n* = 60). **b** Quantitative analysis of lung cancer specimens (*n* = 172) for *SEMA6A* expression. **c** Endogenous expression levels of *SEMA6A* (6A) in different lung cell lines. *6A-FL* was detected by custom manufactured anti-*SEMA6A* antibody from GenScript. Loading control: actin. Positive control: 293T and *6A-FL* overexpressing 293T

Our previous microarray results^19^ showed *SEMA6A* to be significantly downregulated in lung tumor tissue compared to adjacent normal tissue (Table S1). The results were consistently replicated by RT-qPCR (Fig. 1a). Among 172 lung cancer patients, lung cancer tissues from 73.26% of patients were failed to stain with SEMA6A antibody, whereas 26.74% patient samples were stained positively (Fig. 1b). Furthermore, three datasets from Gene Expression Omnibus (GEO)^22–24^ showed similar results (Fig. S1). In addition, endogenous *SEMA6A* was undetectable in several lung cancer cell lines, including A549, H1299, H1975, H441, and H520 (Fig. 1c).

### SEMA6A decreases the growth of lung cancer cells

**Fig. 2:**
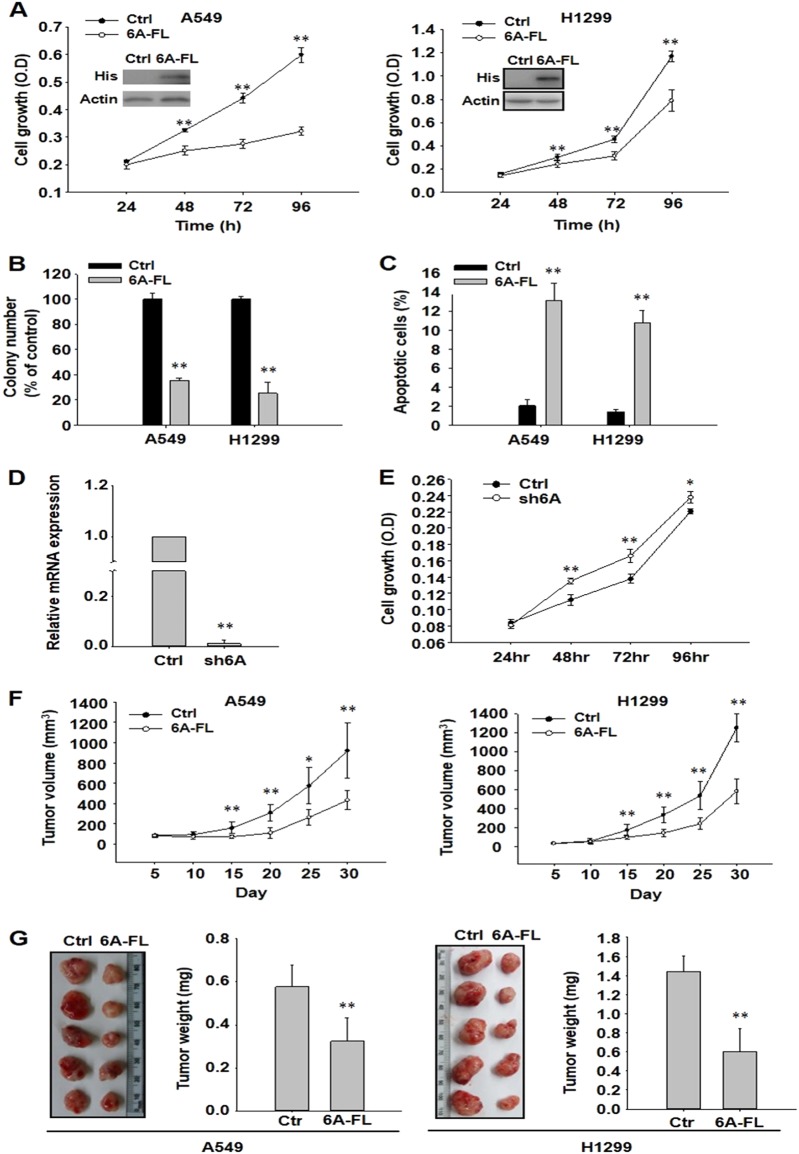
SEMA6A reduces the malignancy of lung cancer cell lines both in vitro and in vivo. **a** Proliferation of A549 and H1299 cells overexpressing *6A-FL* or empty vector (Ctrl). Inset: western blot of His-tagged *SEMA6A*. Loading control: actin. **P < 0.01, *n* = 4. **b** Colony formation of A549 and H1299 cells overexpressing *6A-FL* or Ctrl. Upper, quantitative analysis of colony numbers. **P < 0.01, *n* = 4. **c** Apoptosis analysis of A549 and H1299 cells overexpressing *6A-FL* or Ctrl. **P < 0.01, *n* = 4. **d** Relative mRNA expression level of *SEMA6A* in MRC5 cells expressing *SEMA6A* shRNA and control shRNA lentiviral vectors. **e** The proliferation rate of MRC5 cells with or without *SEMA6A* knockdown. *P < 0.05, **P < 0.01, *n* = 3. **f** Tumor volume in mice transfected with *6A-FL-* or Ctrl-overexpressing A549 (left) or H1299 (right) cells. *P < 0.05, **P < 0.01, *n* = 5. **g** Tumor image, left, and weight, right, from *SEMA6A*-overexpressing A549 and H1299 xenografts. **P < 0.01, *n* = 5

Given the low expression of *SEMA6A* in lung cancer cells, we studied the effects of *SEMA6A* overexpression on cell proliferation, colony formation, and apoptosis in A549 and H1299 cells. Cells with full length *SEMA6A* (*6A-FL*) overexpression had significantly lower cell proliferation (Fig. 2a), fewer colonies (Fig. 2b), and a higher rate of apoptosis (Fig. 2c) compared to cells transfected with empty vector. In addition, silencing of the *SEMA6A* gene in the normal lung fibroblast cell line, MRC5, by shSEMA6A (Fig. 2d) increased cell proliferation significantly (Fig. 2e).

In nude mice, overexpression of *SEMA6A* decreased the growth of A549 and H1299 subcutaneous xenografts (Fig. 2f, g). These results indicate that *SEMA6A* induces apoptosis in lung cancer cells in vitro and reduces lung cancer cell growth in vivo.

### SEMA6A induces apoptosis through its cytosolic domain

**Fig. 3:**
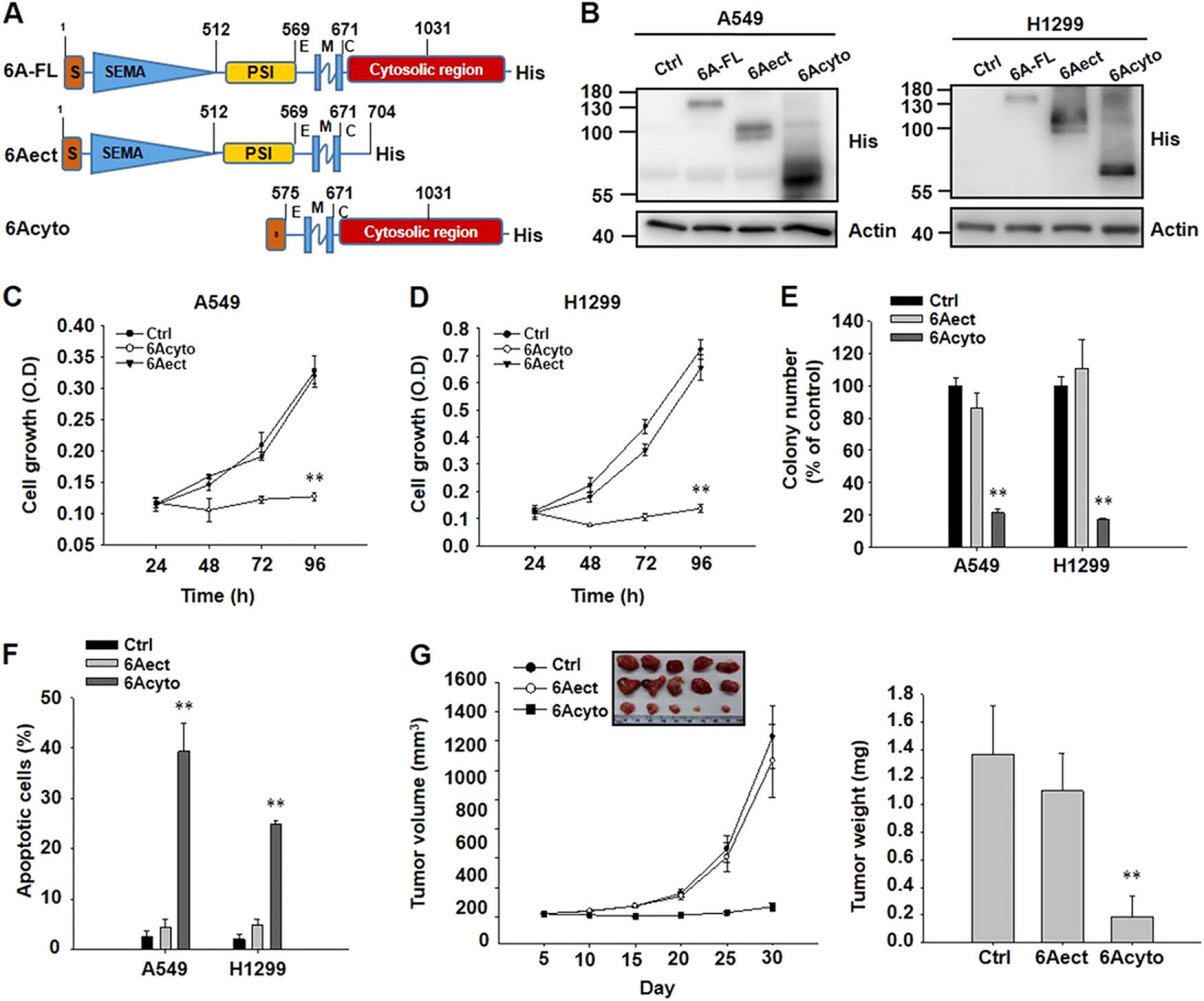
The cytosolic domain of *SEMA6A* induces apoptosis in lung cancer cell lines both in vitro and in vivo. **a** Schematic diagram of the *SEMA6A* constructs. *6A*: full length. *6Aect*: extracellular domain of *SEMA6A*. *6Acyto*: cytosolic domain of *SEMA6A*. **b** Immunoblotting of lysates of A549 and H1299 cells overexpressing *6A-FL*, *6Aect*, *6Acyto*, or empty vector (Ctrl). All constructs were histidine tagged. Loading control: actin. **c** and **d** Proliferation of A549 **c** and H1299 **d** cells overexpressing different truncations of *SEMA6A*. **P < 0.01, *n* = 4. **e** Colony formation of A549 and H1299 cells. **P < 0.01, *n* = 4. **f** Quantitative analysis of the apoptotic A549 and H1299. **P < 0.01, *n* = 4. **g** Tumor volume (left) and weight (right) in mice transfected with *6Aect-*, *6Acyto-*, or Ctrl-overexpressing H1299 cells. Inset: Tumor image from Ctrl-, *6Aect-*, or *6Acyto*-overexpressing H1299 xenografts. **P < 0.01, *n* = 5

Structural and functional analyses were conducted to explore the effects of the extracellular and cytosolic domains and regions of *SEMA6A* in lung cancer cells. The constructs encoding the transmembrane domain with either the intracellular cytosolic domain (*6Acyto*, aa 575–1031) or the entire extracellular ectodomain (*6Aect*, aa 1–704) (Fig. 3a) were overexpressed at similar levels in H1299 and A549 cells (Fig. 3b). Compared to the empty vector cells, the *6Acyto*-overexpressing A549 and H1299 cells significantly decreased cell proliferation (Fig. 3c, d), reduced colony formation (Fig. 3e), and induced more apoptosis (Fig. 3f). In contrast, overexpression of *6Aect* did not significantly alter cell proliferation (Fig. 3c, d), colony numbers (Fig. 3e), and the percentage of apoptotic cells compared to the empty vector-transfected cells (Fig. 3f). H1299 cells with empty vector, *6Acyto*, or *6Aect* overexpression were injected subcutaneously into nude mice. The growth rates and sizes of tumors formed by *6Acyto*-overexpressing H1299 cells were significantly decreased compared to the *6Aect*- and empty vector-transfected H1299 cells in 30 days (Fig. 3g). The tumors with *6Acyto* overexpression were much lighter than those formed from the empty vector- and *6Aect-*overexpressing cells (Fig. 3g). These findings indicate that *SEMA6A*-induced in vivo apoptosis is carried out by its cytosolic domain.

Moreover, we found an unnamed EST clone in the NCBI database (http://www.ncbi.nlm.nih.gov/protein/bah13847.1), BAH13847.1, whose amino acid sequence is highly similar to the transmembrane and cytosolic regions of our *6Acyto* construct (Fig. S2A). Our data demonstrated that this unnamed EST clone could also induce significant apoptosis in lung cancer cells (Fig. S2B). These results suggest that the cytosolic signaling of *SEMA6A* may exist in nature to induce apoptosis in cells.

### The SEMA domain regulates the induction of apoptosis

**Fig. 4:**
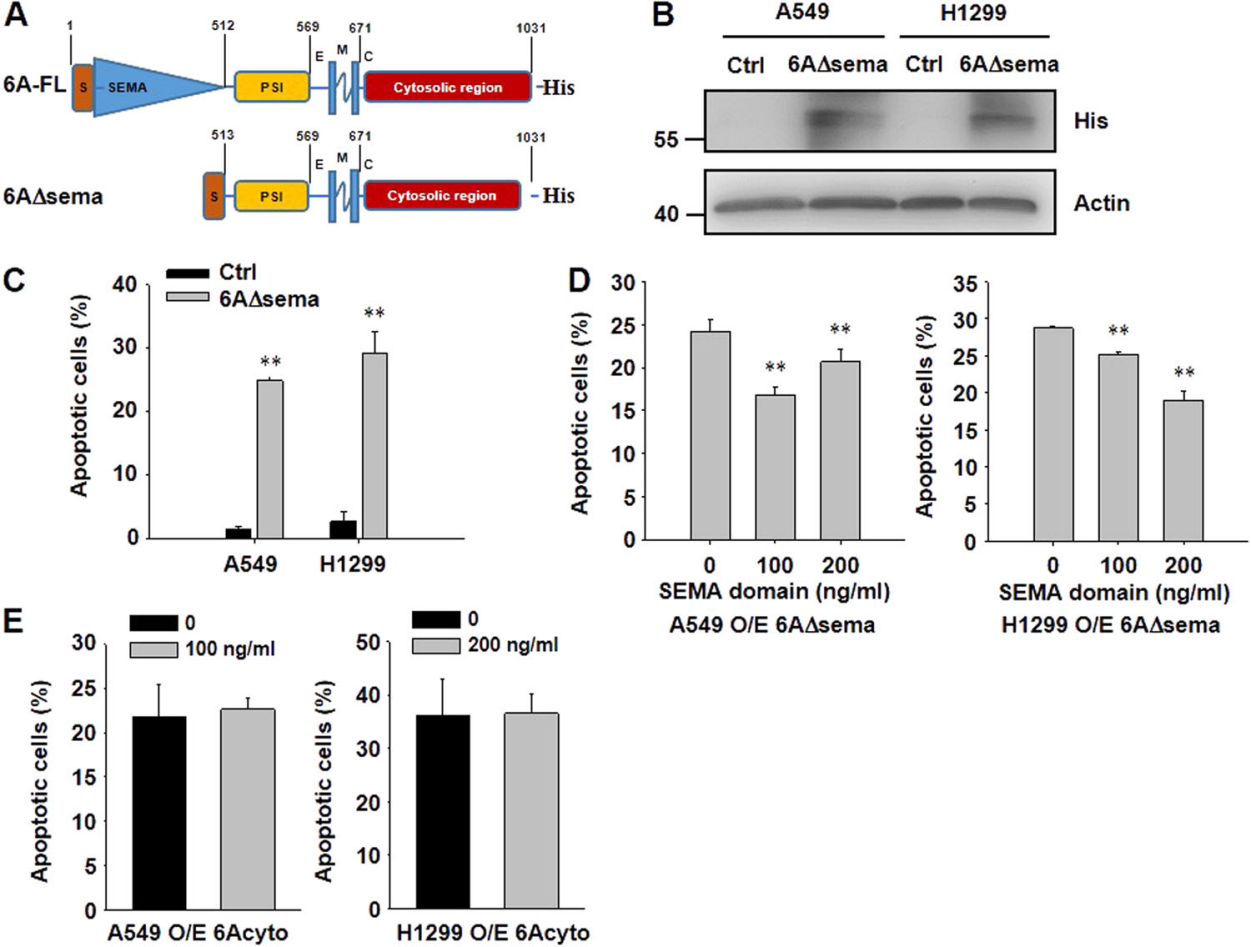
Reintroduction of the *SEMA* domain inhibits the apoptosis induced by *6AΔsema*. **a** Schematic diagram of the *6AΔsema* construct in comparison to *6A-FL*. **b** Immunoblotting analysis of A549 and H1299 cells overexpressing His-tagged *6AΔsema*. Loading control: actin. **c** Percentage of apoptotic A549 and H1299 cells overexpressing *6AΔsema*. **P < 0.01, *n* = 4. **d** Percentage of apoptotic A549 and H1299 cells overexpressing *6AΔsema* after addition of purified *SEMA* domain. **P < 0.01, *n* = 4 per indicated concentration. **e** Percentage of apoptotic A549 and H1299 cells overexpressing *6Acyto* after external addition of purified *SEMA* domain. *n* = 4 for each concentration. *P < 0.05, **P < 0.01

The extracellular region of SEMA6A is dimerized via the SEMA domain to induce plexin-related signaling^25^; however, the other functions of the SEMA domain are still unclear. As *6Acyto* triggered more effects on cell proliferation (Fig. 3c, d) and apoptosis (Fig. 3f) than *6A-FL*, we tested whether the extracellular SEMA domain is required to modulate effects of *SEMA6A* cytosolic signaling in lung cancer cells. Therefore, a construct of *SEMA6A* lacking the *SEMA* domain (*6AΔsema*) (Fig. 4a) was overexpressed in both H1299 and A549 cells (Fig. 4b).

Overexpression of *6AΔsema*-induced apoptosis in 25% of A549 and 29% of H1299 cells, which was comparable to the effect of *6Acyto* overexpression (Fig. 4c), thus establishing that *6AΔsema* and *6Acyto* induce comparable levels of apoptosis. However, when purified SEMA domain (Fig. S3A and B) was added to the cells with *6AΔsema* overexpression, apoptosis was significantly attenuated (Fig. 4d). In contrast, there was no difference in apoptosis observed in *6Acyto*-overexpressing cells upon addition of purified SEMA domain (Fig. 4e). These results indicated that the degree of apoptosis induced by *6A-FL* might be regulated by the SEMA domain.

### The cytosolic domain of SEMA6A interacts with FADD

**Fig. 5:**
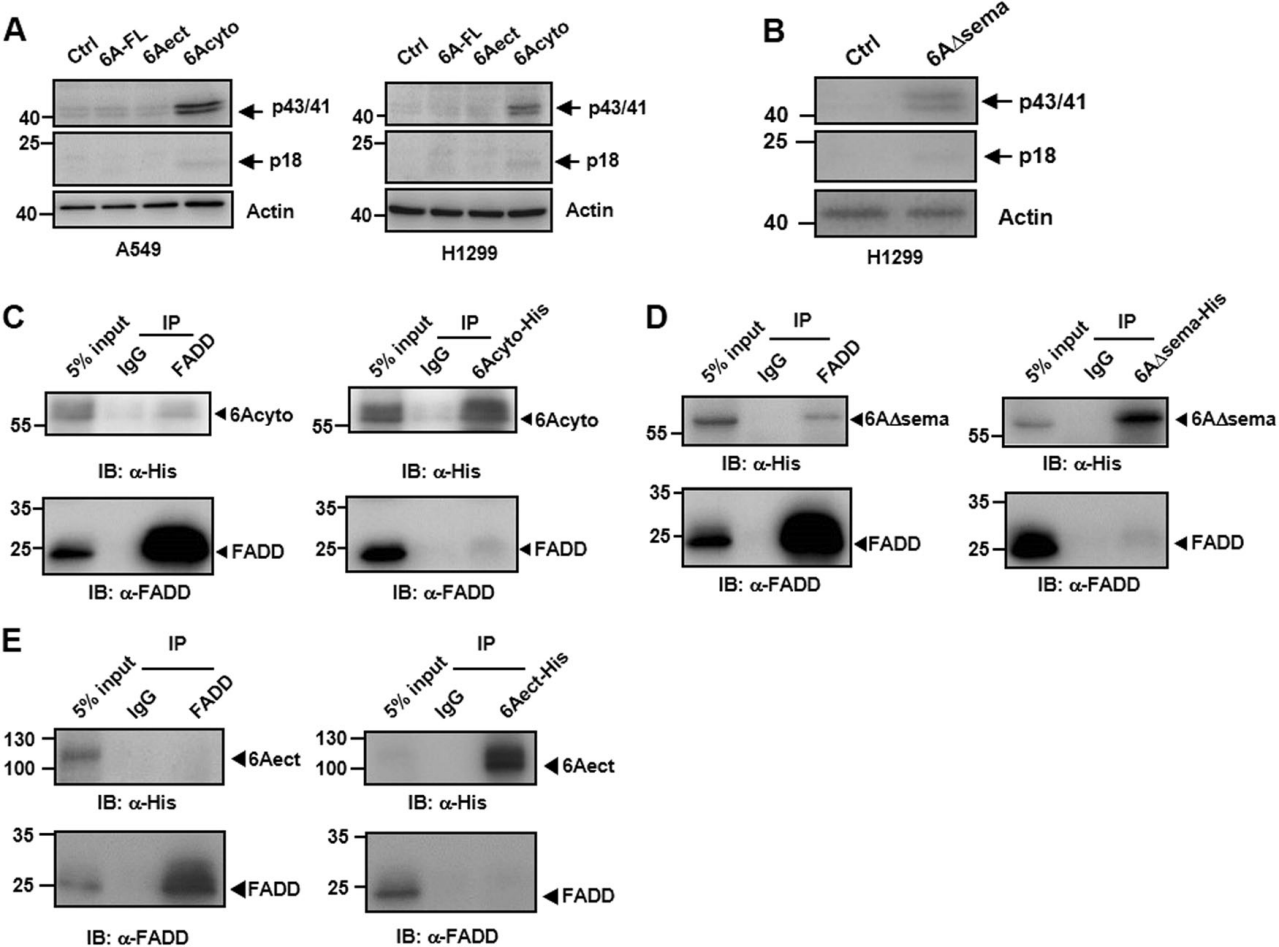
The cytosolic domain of *SEMA6A* binds to FADD. **a** Immunoblot of caspase-8 cleavage products (p43/41 and p18) in A549 and H1299 cells overexpressing constructs of *SEMA6A*. **b** Immunoblotting analysis of p43/41 and p18 in H1299 cells overexpressing *6AΔsema*. Co-immunoprecipitation of FADD and *6Acyto* **c**, *6AΔsema* **d**, and *6Aect* **e** in 293T cells. Input: 5% of non-immunoprecipitated cell lysate. IgG: control IP with isotype-matched IgG antibody from rabbit. The input and immunoprecipitates were subjected to western blot analysis using anti-FADD and anti-His antibodies

The results described so far are the first evidence that the cytosolic signaling mediated by the cytosolic region of SEMA6A can induce apoptosis and is regulated by the SEMA domain in lung cancer cells. We therefore investigated the mechanism by which apoptosis is activated during this signaling. Previous studies have indicated that the activation of DRs such as CD95 or TRAIL receptor (TRAILR)-1 leads to recruitment and activation of FADD, which in turn leads to cleavage of caspase-8, an apoptosis initiator^26–28^. We therefore proceeded to demonstrate that the overexpression of *6Acyto*, but not *6Aect* or *6A-FL*, could activate the cleaved forms (p43/41 and p18) of caspase-8 in H1299 and A549 cells (Fig. 5a); p43/41 and p18 were detected in H1299 cells with *6AΔsema* overexpression as well (Fig. 5b). These results suggest that the membrane-anchored cytosolic domain of SEMA6A might act as a DR by inducing cleavage of caspase-8.

**Fig. 6:**
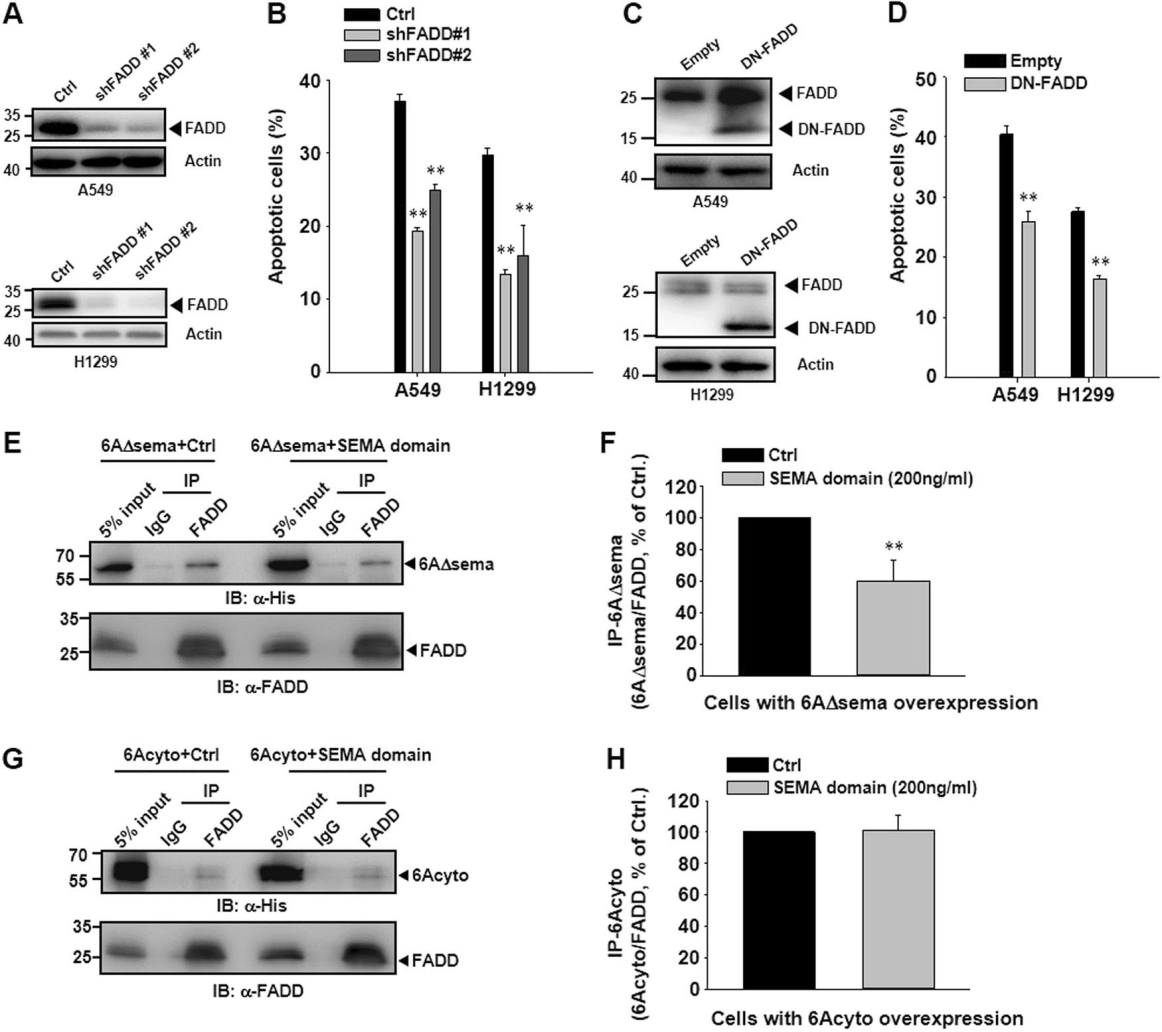
The cytosolic domain of *SEMA6A* induces apoptosis by interacting with FADD. **a** Immunoblotting analysis of silencing FADD in A549 and H1299 cells with two FADD shRNAs (shFADD #1 and #2) and empty lentiviral vector (Ctrl). **b** Percentage of apoptosis in A549 and H1299 cells overexpressing *6Acyto* and transfected with shRNAs against FADD. **P < 0.01, *n* = 3. **c** Western blot analyses of A549 and H1299 cells overexpressing dominant-negative mutant of FADD (DN-FADD). The upper band is endogenous FADD and the lower band is DN-FADD. **d** Percentage of apoptosis in *6Acyto*-overexpressing A549 and H1299 cells with DN-FADD overexpression. **P < 0.01, *n* = 3. Co-immunoprecipitation and quantification of FADD and *6AΔsema* **e** and **f** or *6Acyto* **g** and **h** in the presence of purified *SEMA* domain. **P < 0.01, *n* = 3

To investigate whether the apoptotic pathway induced by the cytosolic domain of *SEMA6A* was similar to these previously identified DRs, *6Acyto*, *6AΔsema*, and *6Aect* were subjected to co-immunoprecipitation with FADD in 293T cells. *6Acyto* was pulled down with FADD (Fig. 5c, left) and, conversely, FADD was also co-immunoprecipitated with *6Acyto* (Fig. 5c, right). Similarly, *6AΔsema* was co-immunoprecipitated with FADD (Fig. 5d, left) and vice versa (Fig. 5d, right). On the contrary, FADD failed to bind to *6Aect* (Fig. 5e) and 6A*-FL* (Fig. S4). Similar results were also observed in H1299 cells (Fig. S5A–C).

### The cytosolic domain of SEMA6A induces apoptosis via interaction with FADD

To demonstrate the requirement of FADD in *6Acyto*-induced apoptosis, we co-transfected *6Acyto* and shRNA targeting FADD in A549 and H1299 cells, which effectively knocksdown endogenous FADD levels (Fig. 6a). Under these conditions, a significant reduction of *6Acyto*-induced apoptosis was observed (Fig. 6b). The same effect was observed when a dominant-negative mutant of FADD (DN-FADD, 80–205) that lacks the death domain^29^ was co-transfected with *6Acyto* in A549 and H1299 cells (Fig. 6c, d). These data suggest that the cytosolic domain of SEMA6A attenuates apoptotic signaling through FADD in lung cancer cells.

Finally, we examined whether the SEMA domain disrupts the interaction of 6Acyto with FADD. Co-immunoprecipitation results showed that the interaction between FADD and 6AΔsema (Fig. 6e, f), but not the interaction between FADD and 6Acyto (Fig. 6g, h), was diminished significantly by the addition of the SEMA domain. These results indicate that the SEMA domain attenuates apoptosis by disrupting the association between FADD and 6AΔsema.

## Discussion

Our study has provided solid evidence supporting a novel role of *SEMA6A* in apoptosis, which can inhibit the growth of lung cancer cells in vitro and in vivo and is regulated by the integral SEMA domain. Previous studies showed that specific protease-induced shedding separates part of the extracellular domain from semaphorins on the plasma membrane, including *semaphorin 4*, and *semaphorin 5*^30–32^. Therefore, the extracellular region of SEMA6A may act as a safety pin, which when removed through proteolytic shedding (“uncapped” SEMA6A*)*, induces apoptosis. In addition, previous structure-based studies indicated that the SEMA6A ectodomain forms a homodimer arrangement to bind plexin A2, inducing the downstream signaling of plexins^14^. Based on such evidence, it can be asserted that the extracellular domain of SEMA6A not only modulates responses for plexin-related signaling, but also functions as a key regulator in SEMA6A cytosolic domain-induced apoptosis in lung cancer cells.

In the *6AΔsema* and *6Acyto* overexpression study, the FADD-associated pathway evidently contributes to *SEMA6A*-related apoptosis. FADD, a critical adaptor protein, has been indicated to activate caspase-8 through binding to the death domains (DDs) of DRs, including CD95/Fas, DR4/TRAIL-R1, and DR5/TRAIL-R2^28,33,34^. Our data showed that 6Acyto was capable of interacting with FADD, thus leading to cleavage of caspase-8. Membrane proteins that induce apoptosis through FADD’s homotypic interaction with their DDs are generally considered to be DRs. As FADD binds to 6Acyto and not to 6A-FL, the 6A-FL could be regarded as a novel inactive type of DR. However, the potential mechanisms that can activate the DR-like SEMA6A need to be further investigated.

Although repression via shFADD and expression of DN-FADD both significantly reduced *6Acyto*-induced apoptosis, the percentage of apoptotic cells remained at more than 15% in both A549 and H1299 cells. Residual endogenous FADD after gene silencing is one possible cause for sustained *6Acyto*-induced apoptosis. In addition to residual FADD expression, the other DR-related apoptotic proteins, such as tumor necrosis factor receptor type 1-associated DEATH domain protein, might be involved in *6Acyto*-induced apoptosis in lung cancer cells.

Overexpression of *SEMA6A* increased apoptosis in both lung cancer cells and normal lung fibroblasts. On the other hand, our data showed that silencing of the *SEMA6A* in MRC5 cells by shSEMA6A increased cell proliferation significantly. In the human body, the total number of cells remains a delicate balance between survival and cell death. A disruption of this balance can trigger the development of cancer^35,36^. Therefore, decreased expression of *SEMA6A* in normal lung fibroblasts might be a potential risk factor for tumorigenesis due to the insufficient activation of apoptosis. These findings suggest that the expression level of *SEMA6A* may be a prognostic biomarker for lung cancer patients.

In conclusion, we have provided the first evidence that the extracellular SEMA domain of *6A-FL* appears to function as an attenuator of apoptosis via association of its cytosolic region with FADD in lung cancer cells. These data assert that wild-type SEMA6A is just like a “capped protein” whose ability to induce apoptosis is limited by the cap, i.e., the SEMA domain. Compared to the well-known DRs that can trigger autocrine death or paracrine suicide^37^, *SEMA6A* uses a novel mechanism to regulate ligand-independent apoptosis through its SEMA domain.

## Materials and methods

### Quantitative RT-PCR

After reverse transcription of total RNA from patients’ tissues, qPCR was processed using an ABI7300 system (Applied Biosystems, Foster, CA) with SYBR Green (Roche, Basel, Switzerland) according to the standard protocols. The forward and reverse primers of *SEMA6A* were 5′-GCTACACTTTGCTGGGGCTAA-3′ and 5′-CGGCTTTGGGTCTTTGGATTG-3′, respectively. The collection of patient samples was described in our previous study^18^. The institutional review boards of National Taiwan University Hospital and Taichung Veterans General Hospital both approved the sample acquisition and its subsequent use (IRB #200610015R).

### Microarray data analysis

Microarray datasets from GSE19804, GSE18842, GSE4079, and GSE19188 were used to identify the expression level of *SEMA6A* in lung cancer patients. The intensity data of *SEMA6A* in these studies were analyzed by Partek (Partek, Chesterfield, MO) to obtain mRNA expression levels. Probe-level data of *SEMA6A* were preprocessed through background correction, quantile normalization, and summarization, using robust multi-array average analysis. The paired *t*-test (*P*-value<10^−16^) was used to identify differentially expressed *SEMA6A*.

### Cell culture

Lung cancer cell lines (A549, H1299, H1975, H441, and H520) were purchased from Bioresource Collection and Research Center (BCRC, Hsinchu, Taiwan). Lung cancer cell lines H1299 and A549 with exogenous luciferase (Luc) activity were kindly provided by Dr. P.W. Hsiao. All cell lines were cultured following the protocols at BCRC and authenticated by STR-PCR profiling at BCRC.

### Immunohistochemical staining

A total of 172 lung cancer samples collected from National Taiwan University Hospital (Taipei, Taiwan) and Taichung Veterans General Hospital (Taichung, Taiwan) were used for the examination of *SEMA6A* expression. The paraffin-embedded tissues were de-paraffinized and reactivated as described earlier^38^, after which the tissue sections were incubated with normal goat serum (dilution 1:500; Dako) at room temperature for 2 h followed by primary *SEMA6A* antibody (dilution 1:50; Cat. #AP2740b; Abgent, San Diego, CA) at 4 °C overnight. Streptavidin- and biotin-labeled secondary antibodies were used for staining.

### SEMA6A, 6AΔsema, 6Acyto, and 6Aect constructs

The full length *SEMA6A* (*6A-FL*) cDNA was generated from normal foreskin fibroblasts, Hs68, using RT-PCR. The cDNAs of the extracellular ectodomain (*6Aect*, aa 1–704), the intracellular cytosolic domain (*6Acyto*, aa 575–1031), and the *SEMA* deletion mutant (*6AΔsema*, aa 513–1031) were produced from the *6A-FL* sequence. The primers are listed in Table S2. The cDNA fragments were then sub-cloned into the lentiviral expression vector pCDH-CMV-MCS-EF1-puro (System Biosciences), and *6A-FL* and all domain constructs were fused with a 6X His-tag at the C-terminus of the expressed protein.

### Virus production and cell infection

HEK293T cells (4 × 10^6^ cells) were co-transfected with transfer vector (pCDH-CMV-MCS-EF1-puro; 8 μg), packaging plasmid (psPAX2; 6 μg) and envelope plasmid (pMD2G; 2 μg) using TransIT^®^-2020 (Mirus Bio, Madison, WI). The supernatants containing infectious particles were collected, aliquoted, and stored at −80 °C at 48 h and 72 h post-transfection. The virus was added to 4 × 10^4^ cells, and the cells were centrifuged at 2500 rpm for 60 min at room temperature.

### Western blotting

Proteins were extracted using RIPA lysis buffer (Millipore, Billerica, MA) containing protease inhibitor cocktail, 10 mM β-GP, and 5 mM Na_3_VO_4_. Protein from lysate was separated by 10% SDS-PAGE and transferred onto a nitrocellulose membrane (Millipore). The blots were probed overnight at 4 °C with primary antibodies against SEMA6A (custom manufactured antibody from GenScript), His-tag (Cat. #05–949; Millipore), β-actin (Cat. # ABT264; Millipore), and cleaved caspase-8 (Cat. #9496S; Cell Signaling). After incubation with the appropriate HRP-conjugated secondary antibodies, HRP activity was visualized by an enhanced chemiluminescence system (UVP, Upland, CA).

### MTT assay

Cells were seeded at 2 × 10^4^ cells/well on a 24-well plates and then incubated at 37 °C for 3.5 h with 100 μL of 5 mg/mL MTT (3-(4,5-dimethylthiazol-2-yl)-2,5- diphenyltetrazolium bromide, SIGMA, Saint Louis, MO) at specific time points. Subsequently, the cells were shaken with 400 μL MTT solvent (4 mM HCl mixed with isopropanol) at room temperature for 15 min. Absorbance was read at 590 nm with a reference filter of 620 nm by ELISA reader (PerkinElmer, Waltham, MA).

### Annexin V analysis for apoptosis

Cells were stained with propidium iodide and annexin V following the manufacturer′s instructions (BD, San Jose, CA). A minimum of 10,000 cells in each sample were detected by a Cytomics FC500 cytometer (Beckman, Brea, CA). Cell apoptosis was analyzed by WinMDI software.

### Clonogenic assay

Cells were seeded in a 6 cm dish at 200 cells/dish and incubated for 10 days. Colonies were fixed with 75% alcohol, stained with 20% crystal violet solution, and manually counted. Colony formation was described as follows: colony number/(number of cells seeded × plating efficiency), where plating efficiency was equivalent to the colony number divided by the number of cells with empty virus infection.

### Xenograft tumor models

Male nude mice, 4–6 months old, were purchased from the National Laboratory Animal Center (Taipei, Taiwan). The lung cancer cells (3 × 10^6^) were re-suspended in 100 μL medium and mixed with Matrigel at a 1:1 ratio by volume and then injected subcutaneously into the right flank of each mouse. Five mice were randomly divided into each group. After injection, a Vernier caliper was used to measure the tumor size weekly. Tumor volumes were calculated by the formula V = 1/2 (Length × Width^2^). At the sixth week, the mice were weighed and then killed. The number of mice was determined by pilot experiments between the mice injected with 6A-FL-overexpressing and control lung cancer cells. The experiments were performed in compliance with the protocol (IACUC-20130394) of the Research Ethics Office at National Taiwan University.

### Purification of SEMA protein

HEK293T cells were transfected with the vector (pcDH-CMV-MCS-EF1-puro) for *SEMA*-*His* by the TransIT-2020 reagent (Mirus). After 3 days, the supernatants were applied to a 3 mL Ni-containing resin. Protein was eluted with 300 mM imidazole, 300 mM NaCl, and 20 mM sodium phosphate, pH 7.4. The eluted sample was passed through a 0.45 μm filter and applied to Sephacryl s-100 h gel filtration columns (GE Healthcare, Little Chalfont, UK) on an ÄKTA FPLC system (GE Healthcare) at a flow rate of 0.5 mL/min in PBS. Eluate was collected as 40 fractions and maintained at 4 °C. Each fraction was assessed for protein by immunoblotting analysis with anti-His mAb (1:5000, Millipore).

### Co-immunoprecipitation assay

After 48 h overexpression of 6X His conjugated truncations of SEMA6A, cells were lysed with ice-cold Pierce^®^ IP lysis buffer (Thermo Fisher, Waltham, MA). Cell lysates were incubated with rabbit anti-SEMA6A (custom manufactured antibody from GenScript) or rabbit anti-FADD antibodies at 4 °C for 16 h. Cell lysates were incubated with normal rabbit IgG (Millipore) as control. Then, magnetic beads (Thermo Fisher) were added to cell lysates and incubated overnight at 4 °C for immunoprecipitation. Beads were washed three times with wash buffer, boiled in sample buffer, and analyzed by western blotting.

### Silencing of FADD

shFADD clones, TRCN0000332992, and TRCN0000332994, were obtained from the National RNAi Core Facility (Academia Sinica, Taiwan). The production of lentivirus for silencing of FADD was described above.

### Statistical analysis

The investigators were randomly allocated and blinded to the different groups during collection of results of each experiment. A two-tailed Student’s *t*-test was applied to all the data in this study. The variance is similar between the groups in the same experiment. Differences were considered to be significant if the *P*-value was <0.05. All values in the text and figures are presented as mean ± standard deviation. The sample sizes are described in the figure legends.

## Electronic supplementary material

Supplementary Figure 1

Supplementary Figure 2

Supplementary Figure 3

Supplementary Figure 4

Supplementary Figure 5

Table S1

Table S2

## Data Availability

All data generated or analyzed during this study are included in this published article [and its supplementary information files].
