# Supplementary figures and images for: The extracellular SEMA domain attenuates intracellular apoptotic signaling of semaphorin 6A in lung cancer cells
Source: Oncogenesis. 2018 Dec 5;7(12):95. doi: 10.1038/s41389-018-0105-z (PMC6281666; doi:10.1038/s41389-018-0105-z)

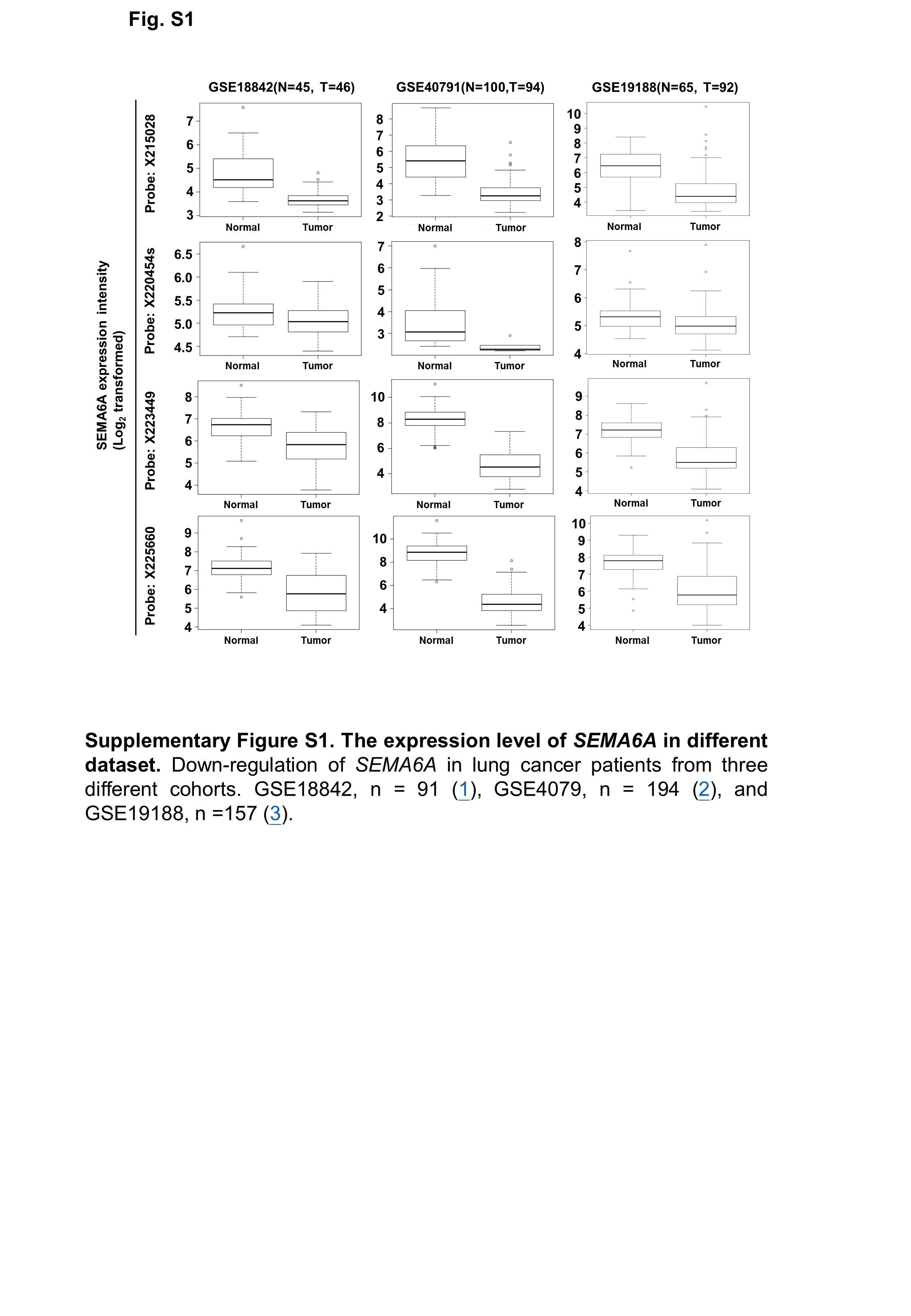

Supplement: Supplementary file 1 — Supplementary Figure 1 [file 41389_2018_105_MOESM1_ESM.jpg]

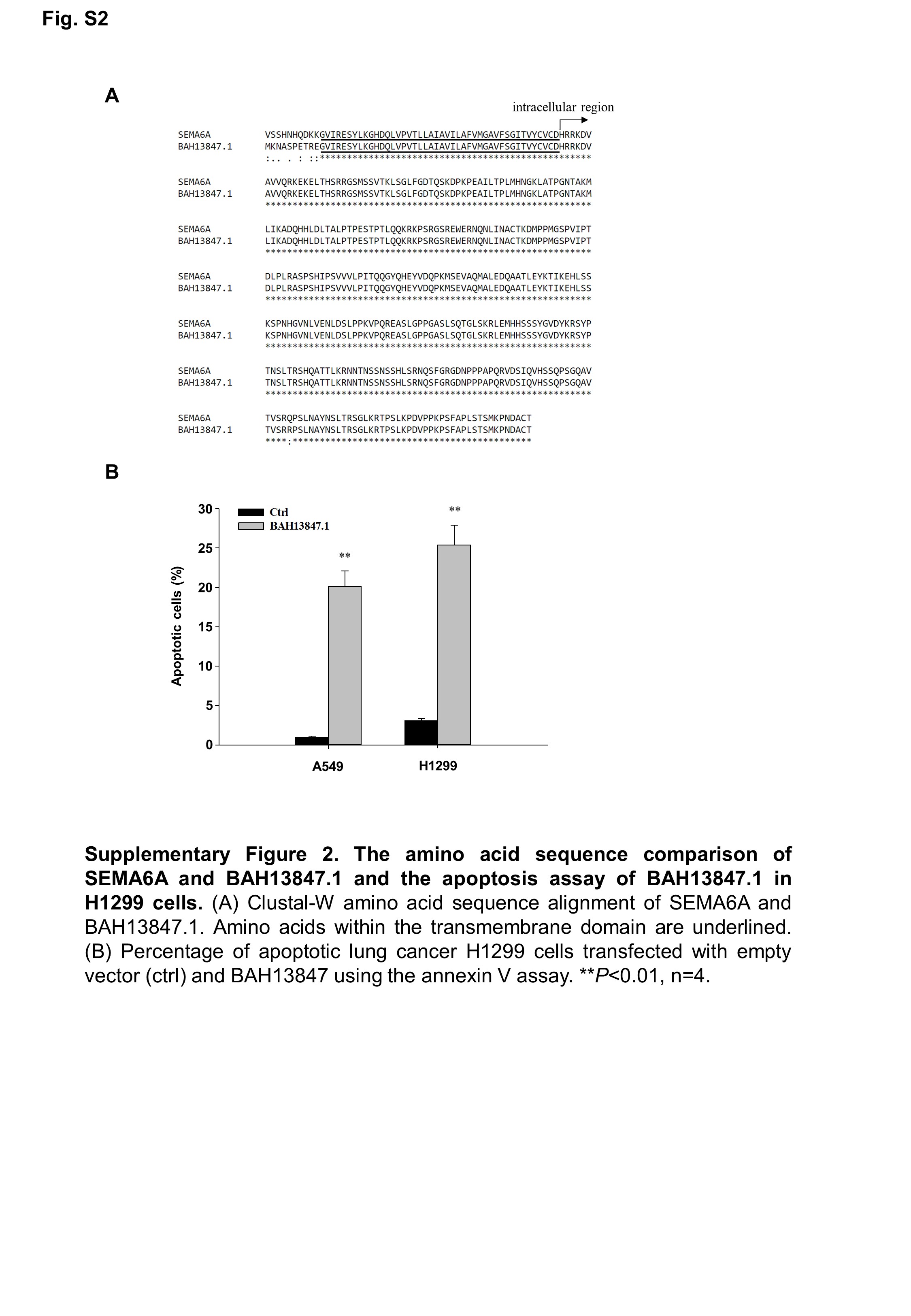

Supplement: Supplementary file 2 — Supplementary Figure 2 [file 41389_2018_105_MOESM2_ESM.jpg]

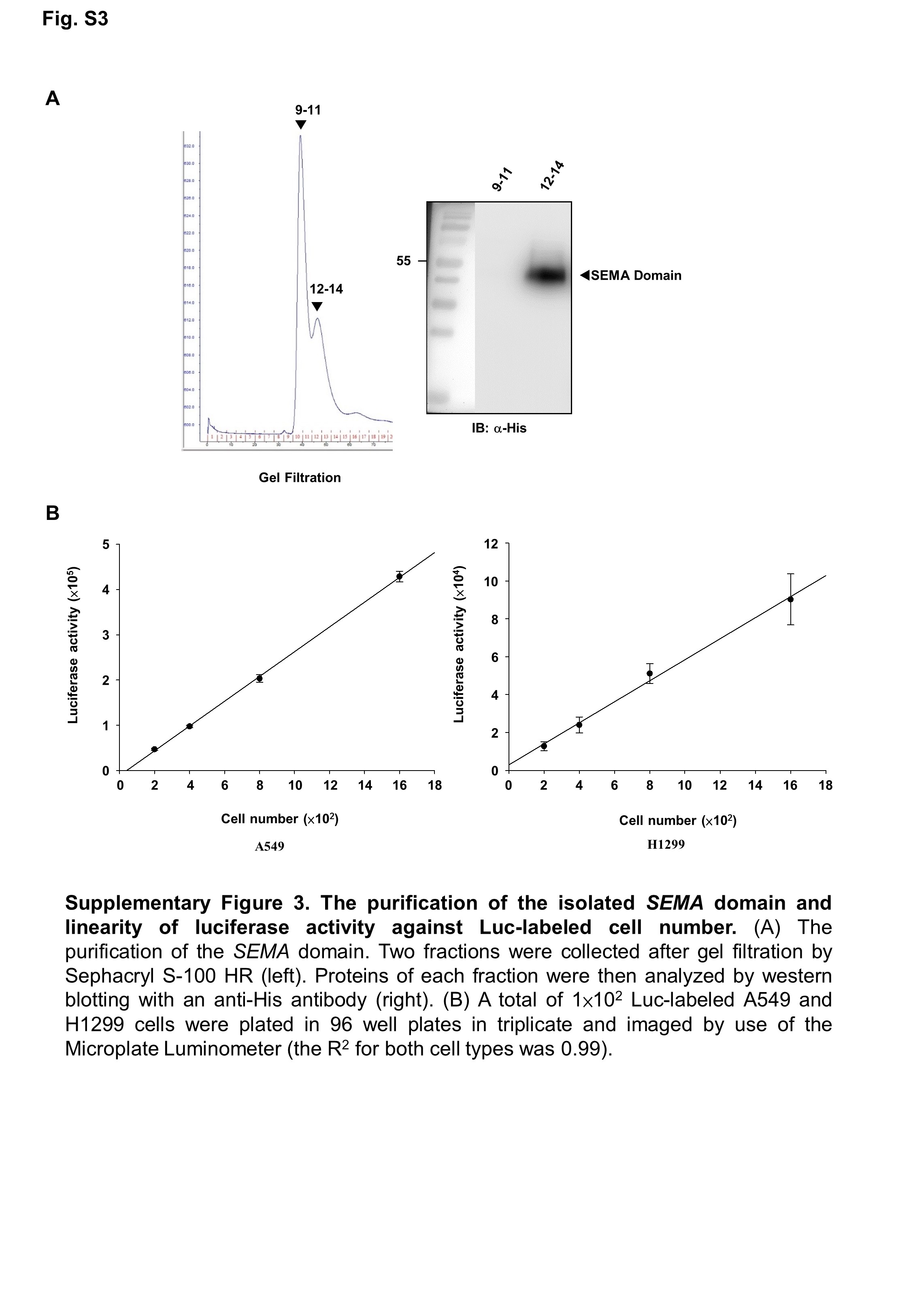

Supplement: Supplementary file 3 — Supplementary Figure 3 [file 41389_2018_105_MOESM3_ESM.jpg]

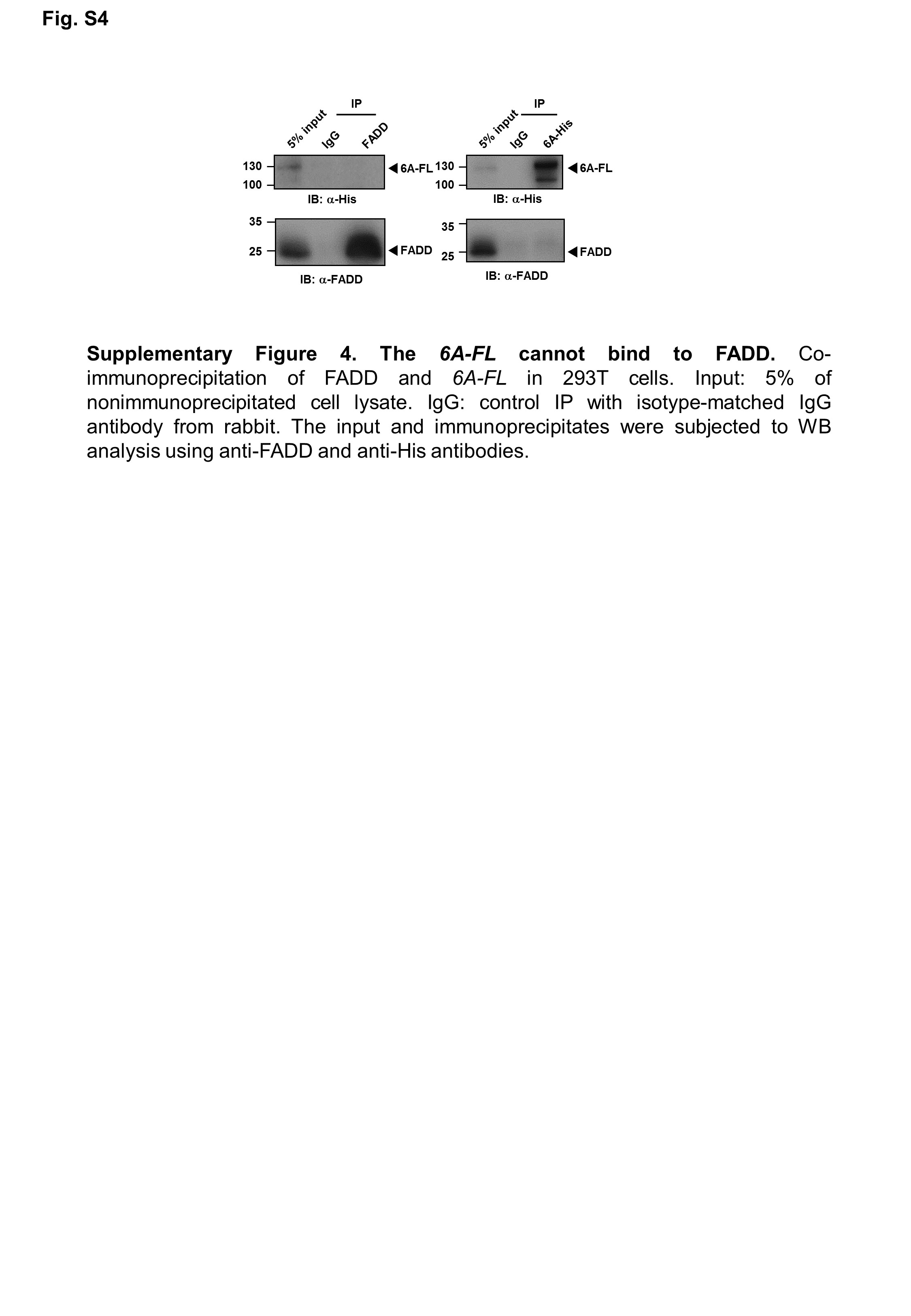

Supplement: Supplementary file 4 — Supplementary Figure 4 [file 41389_2018_105_MOESM4_ESM.jpg]

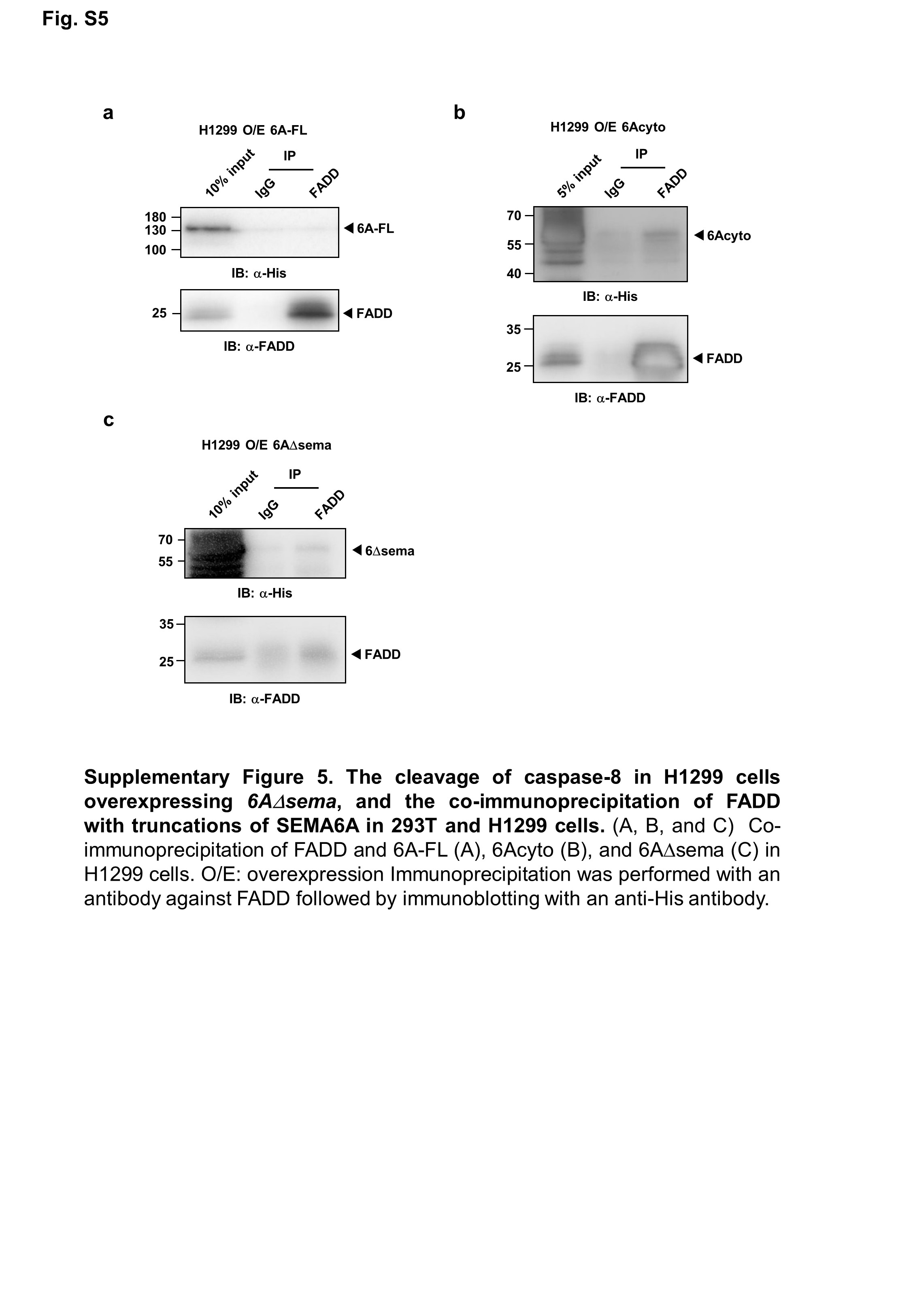

Supplement: Supplementary file 5 — Supplementary Figure 5 [file 41389_2018_105_MOESM5_ESM.jpg]
