# Supplementary material for: The extracellular SEMA domain attenuates intracellular apoptotic signaling of semaphorin 6A in lung cancer cells
Source: Oncogenesis. 2018 Dec 5;7(12):95. doi: 10.1038/s41389-018-0105-z (PMC6281666; doi:10.1038/s41389-018-0105-z)
Supplement: Supplementary file 6 — Table S1 [file 41389_2018_105_MOESM6_ESM.pdf]

Table S1

Probe ID	Gene symbol	Cancer/Normal ratio	P-value
210528_at	SEMA6A	0.357*	9.33×10^{-13}
2200454_s_at	SEMA6A	0.700*	6.85×10^{-10}
223449_at	SEMA6A	0.318**	7.44×10^{-17}
225660_at	SEMA6A	0.288**	9.87×10^{-18}

Down-regulation of SEMA6A in lung adenocarcinoma using Affymetrix GeneChip® Human Genome U133 Plus 2.0 expression arrays. *SEMA6A* was analyzed from 60 pairs of cancerous and normal tissues, and the ratio of expression is shown. *: $P < 10^{-10}$ and **: $P < 10^{-13}$.
