# Supplementary material for: The extracellular SEMA domain attenuates intracellular apoptotic signaling of semaphorin 6A in lung cancer cells
Source: Oncogenesis. 2018 Dec 5;7(12):95. doi: 10.1038/s41389-018-0105-z (PMC6281666; doi:10.1038/s41389-018-0105-z)
Supplement: Supplementary file 7 — Table S2 [file 41389_2018_105_MOESM7_ESM.pdf]

**Table S2**

| Gene name | Primer[1] | Primer sequence |
|---|---|---|
| **Wild type SEMA6A** | | |
| *6A-FL* | Forward | ATGAGGTCAGAAGCCTTGCT |
| | Reverse | TGTACACGCATCATTG |
| **Different truncations of SEMA6A** | | |
| *6Aect* | Forward | ATGAGGTCAGAAGCCTTGCT |
| | Reverse | CTTGGTGACGCTG |
| *6Acyto* | Forward[2] | AATGGGCATTCCAGT |
| | Reverse | TGTACACGCATCATTG |
| *6AΔsema* | Forward[2] | GGCCGGTGTGAACGACATG |
| | Reverse | TGTACACGCATCATTG |
| *6Asema* | Forward | ATGAGGTCAGAAGCCTTGCT |
| | Reverse | AAGGGGAACC |
| **Dominate negative inhibitor** | | |
| *DN-FADD* | Forward | ATGGACGACTTCGAGG |
| | Reverse | TCAGGACGCTTCG |

1. All forward primers are conjugated with star codon, kozak sequence, and BamHI cutting site. Except DN-FADD, the reverse primers are conjugated with 6 × His, stop codon and BamHI cutting site.
2. The forward primers are conjugated with signal peptide which belongs to SEMA6A.
